# Observations on the Poisonous Properties of the Sulphate of Quinine

**Published:** 1847-10

**Authors:** William O. Baldwin

**Affiliations:** Montgomery, Alabama


ART. II—. *Observations on the Poisonous Properties of the Sulphate of Quinine.* By WILLIAM O. BALDWIN, M. D., of Montgomery, Alabama. 8vo., pp. 22.

The toxicological properties of medicinal remedies cannot be too diligently studied, and should never be lost sight of by the practitioner of medicine. For want of due attention to this subject, there is reason to believe that our art, not only often fails of attaining its legitimate purposes, but in some instances, produces effects the reverse of those which are desired. The medicinal character of every powerful remedy has a certain limit, as respects quantity, in either direction. In too minute doses it is not only innocuous, but devoid of any useful effect; and, thus administered, will do no good, although, by displacing means which would be calculated to exert a beneficial efficacy, it may indirectly do an incalculable injury. This remark applies to Homœopathy when its remedies are honestly administered in infinitessimal quantities. It is hardly necessary to caution medical practitioners against falling into this absurdity. But that there is a tendency to an extreme in the other direction, and that a large proportion of the profession have erred, and are still liable to err in the excessive use of powerful medicinal agents, must be admitted. To this source is undoubtedly to be attributed, in some measure, the transient popularity of the homœopathic delusion.

It is never to be forgotten, that when any medicine is administered in quantities beyond the limit of its medicinal character, it becomes truly poisonous, and under such circumstances, must exert, more or less, injurious influences. In connection with this important principle we will submit another, which, in our opinion, the practitioner will do well never to forget:—it is, that a remedy, even when used as a remedy, (i. e., within appropriate limits) if it does not exert a good influence, will necessarily do more or less harm. If it have any potency whatever, it must be potent for good or evil. There is scarcely an exception to this rule. There are no powerful remedies which, if they do not produce a beneficial effect, are harmless. We will waive for the present the inquiry in far far this rule is overlooked by medical practitioners, and proceed to the publication before us, the title of which is prefixed to this article.

Dr. Baldwin's paper on the poisonous properties of Quinine originally appeared as a communication in the American Journal of the Medical Sciences. The subject is one of interest and importance. The attention of the profession has been of late years directed to the employment of quinine in much larger doses than were formerly considered judicious, or even safe. This remedy has also been employed under circumstances which formerly were thought to render it, not only useless, but pernicious. We need not now go into a digest of the facts which have accumulated respecting the advantages of using the remedy with greater freedom and promptness than was the custom but a few years ago. Suffice it to say, that its remedial efficacy has been vastly extended, and it has been shown to furnish available resources far exceeding the most sanguine expectations of those who first ventured on its use. But this, like every other remedy, has its limit, beyond which it ceases to be, properly speaking, a remedy, and becomes a poison. The tendency of the human mind is to extremes. No sooner was it established that large doses of this valuable specific could be safely administered, under almost every variety of circumstances, and with signal success, than a natural enthusiasm led some to prescribe it in enormous quantities. That in this way it has done injury we cannot but believe. It is, then, a point of no little practical importance, to determine the limits of its remedial character, and to ascertain, if possible, when it ceases to be a medicine, and becomes a poison. The researches of Dr. Baldwin have some bearing on this point, and we therefore deem them sufficiently important to present an analysis of them, referring those of our readers to whom the American Journal is accessible, to the April No., 1847, of that valuable periodical for the entire article.

Dr. B. first reports a case of convulsions, blindness, and death, as the supposed effects of quinine, the patient being a child six years of age. The quantity which appeared to produce the fatal result was four grains, repeated in three hours. It had, however, been administered at regular intervals, but in lesser quantities, for two days previous. The symptoms immediately following the two last doses were *extreme restlessness, dilitation of the pupils, blindness, and convulsions*. In a second case, the details of which are given, sixty eight grains were introduced into the system in the space of twenty and a half hours, the patient, judging from the symptoms, being affected with fever of a continued type. The effects attributed to the quinine in this case, are *restlessness, tremors, slow and irregular breathing, dilitation of the pupils, blindness and convulsions*. The patient recovered after a protracted illness.

These cases are not sufficient in number to authorize any positive deductions concerning the character of the toxical effects of quinine; and there is some room for skepticism as to the phenomena attributed to this remedy being in reality legitimate effects of it, disconnected from the disease. Several cases, however, have been recorded by different observers in which similar accidents attending its employment occurred. The writer refers to the article on "Quinquina" in the *Dictionnaire de Medicine*, for a collection of such cases. This, together with the fact that in experiments upon inferior animals the same train of results seemed to be produced, renders the presumption very strong that the symptoms detailed in two cases reported by Dr. B. are fairly attributed to the poisonous operation of the drug under consideration.

The animal selected by Dr. Baldwin for his experiments was the dog. The toxical effects, as manifested in this animal, are concisely presented in the following extract:—

"*Symptoms* which followed the ingestion of large doses of quinine into the stomachs of dogs:—*restlessness* generally preceded all other symptoms, as was indicated by the animal changing its position often, and constantly moving from place to place. *Vomiting*, or, in those cases where the œsophagus was tied, efforts to vomit succeeded. *Purging* was noticed occasionally, but in no instance except where the medicine was taken by the stomach. Then came on *muscular agitation*, or *tremulous movements* of the body and extremities, with a *constant motion of the head*, resembling somewhat *paralysis agitans*, In attempting to walk, the dog would totter from side to side and fall, or if he maintained his feet, would walk in a direction different from the one which he seemed to desire. When under the full operation of the poison, the power of locomomotion, or even the power of standing in the erect position was lost altogether, the extremities apparently *completely paralysed.* This state was accompanied with more or less *excitement of the vascular system;* the pulse increasing in frequency and rising from 110 to 160, and in one instance even as high as 240 per minute. *Great oppression of the breathing* was present, and sometimes *frothing at the mouth.* The *dyspnœa* in all instances was excessive, sometimes panting, at others *slow* and *labored*, resembling in a most striking manner an acute attack of asthma; countenance expressive of great distress and anxiety. The *pupils of the eye were invariably dilated*, and generally to an enormous extent, leaving but a small ring of the iris perceptible, and *vision*, as well as could be judged, *was entirely lost. Convulsions* were observed in every case (except one), which was watched to its termination, where the dose given was sufficient to produce death, and in one or two instances where the medicine failed to produce this result. *Furious delirium* was present in one case, as was manifested by the dog barking and biting at everything about him. Sometimes a *profound coma* would ensue, accompanied with slight *muscular agitation*, *slow* and *heavy breathing*, terminating in death in a very few minutes after the poison had been taken, and in a few instances the subject seemed as if stunned by some sudden and powerful blow or violent fit of apoplexy. This latter effect, however, was only observed when it was given to young dogs (half grown and under) through the jugular vein or peritoneum. Its effects upon puppies seemed to be *proportionately* much greater than upon dogs fully grown.

The time required to produce death varied very greatly with the quantity given and the age of the subject, as well as the mode and manner of its administration, and some instances it varied considerably when the dose, mode, and all other circumstances of its administration were suppposed to be equal; for, whilst in some instances fifteen or twenty grains produced the uniform and peculiar train of toxical symptoms, succeeded by death in a very short time; in other instances is required these quantities doubled and repeated until 120 grains had been taken, and much longer time to produce the same results. This fact is in accordance with my experience relative to its remedial action upon the human subject, showing that it is governed more, perhaps, in its modus operandi by inherent idiosyncrasies, or created predispositions, than any other remedy. The modes of giving it adopted were by the stomach, the cavity of the abdomen, and by the jugular vein. When given by the stomach it produced vomiting, and was thrown back generally before a sufficient amount to produce death could be absorbed. By dissolving and largely diluting it with water, a sufficient quantity was absorbed to produce death, in this manner, in one instance. In almost all of the experiments with it by the stomach, however, the œsophagus was ligatured. When dissolved and given by the *stomach*, its first effects were observable in about twenty minutes, sometimes shorter or longer, and death resulted in from one to thirty-six hours, usually in four or six. An empty stomach facilitated its operation greatly. When injected into the *peritoneum* in full doses (40 grs.) its effects were appreciable in from four to six minutes, and death occurred in from thirteen to thirty minutes. When injected into the *jugular vein* (in giving it by this mode great care was taken to prevent the admission of air), its first effects were manifest in a space of time so short as to be almost inappreciable; not more than a few seconds after the nozzle of the syringe was withdrawn, and death occurred in one or two minutes. In all instances, except one, the quinine was dissolved in water by the addition of sulphuric or other acid in quantities barely sufficient for this purpose.*

When the experiments went far enough to produce amaurosis, short of death, the vision was regained after a time. In one instance the dog remained *totally blind* for two weeks, and afterwards regained his vision slowly. This is also a feature in the second case reported in the commencement of this article. The man regained a very useful degree of vision after a short time. From these, as well as other cases of the kind reported, it would seem that amaurosis from this cause is not likely to be permanent.

Though it operated much more promptly when injected into a vein or the peritoneum, yet I did not observe that it operated with more power or force: that is, I did not discover that a given quantity administered in this way would produce death more certainly than when given on an *empty* stomach. 28 grains injected into the cavity of the abdomen in one instance, and 20 grains injected into the jugular vein in another, failed to produce death, yet these quantities *did* produce death in other instances, as well when given by the *stomach, or by these modes.*

*The post-mortem appearances* were equally uniform with the symptoms before death. The most prominent and characteristic appearances were the *dark, fluid* and *defibrinated* condition of the *blood* and the *congested* state of the paranchyma of the *lungs,* resembling very much *red hepatization.* The vessels of the membranes of the brain were engorged, so also were the liver and kid-

---

* In one instance where it was made into a bolus and enveloped in a slice of bacon and introduced into the stomach, vomiting occurred in twenty minutes, and a large portion of the medicine returned. Except that it did not produce death, its effects did not differ in this from those observed in other instances where it was dissolved and given by the stomach.

neys in a few instances. The stomach and bowels were vascular and highly injected in patches. The membranes of the spinal cord were more or less vascular, and, in one instance, a semi-fluid coagulum of blood was found in the upper half of the theca vertebralis. This was probably owing to the subject being very young, and the convulsions being much more violent and frequent than in any other instance.

The effects of remedies administered experimentally to inferior animals, should, in our opinion, be very cautiously received as representing those which occur in the human subject. But to some extent this analogical reasoning is undoubtedly legitimate. The correctness of the conclusion that certain *post hoc* phenomena are *propter hoc*, is confirmed, if phenomena of a similar character, in so far as a comparison can be instituted, may be produced in an animal at will. In general, with regard to the evils which may arise from the quinia, a just inference is, if twenty or thirty grains will kill a dog in the space of a few hours, that fifty, sixty, seventy, or more grains taken into the human stomach at once, after short intervals, cannot be devoid of all danger. The writer's reflections on this point seem to us to be just:—

Thus it seems clear that quinine is a *poison*, and one which may be made *directly fatal to life*, and if these experiments upon the dog, *in themselves* are not conclusive of that fact, which the concurrent testimony of toxicologists would justify us in believing, they at least become so when it is remembered that the symptoms which its exhibition gave rise to, are not only strongly corroborated by, but were almost identically the same with those observed in the human subject, in the few instances where poisoning from this substance is known to have been produced. There is not a symptom noticed in these experiments which has not, at one time or other, been observed in its operation upon the human subject, and the two cases of poisoning in the human subject reported in the commencement of this article, where the striking and peculiar assemblage of symptoms which followed its administration was so completely identical with those observed in the dog most clearly established the fact that the manifestations of its *posionous operation*, at least upon the dog, are identical with those observed in the human subject, or at any rate, do not differ more than they do in different instances on "man and man."

Its operation as a poison, as well as a remedy, is certainly peculiar, and it seems difficult to assign it to any particular class of poisons, differing in some respects from all of them. It appears to resemble in its action, more closely than any other, those of the "*second class*" of Orfila, or the class of "narcotic poisons." It does not seem to possess any hypnotic properties; in this it differs from most of the substances included under this head. I do not mean to touch the much agitated question of the mode of its remedial operation, but desire to speak of its poisonous action only; and, on this head will only add, farther, that its operation seems to be principally upon the nervous system, as is clearly demonstrated in the derangement of the senses of vision and hearing, and respiratory functions, as also in the general muscular agitation, convulsions, &c. As it has been detected in the urine there can be no doubt but that it enters and mixes with the circulating masses of the body, and through this means exerts a *direct* influence upon the nervous system, which, as we have seen, is *eminently* excitant when given in quantities calculated to destroy life."

To illustrate the excessive boldness with which the Quinine has been used by some practitioners, the following quotation is given from an article by Dr. Dickson, in the Southern Journal of Medicine :

" A medical friend in Alabama assures us that he had administered *thirty grains* of the *solution* of quinine *every hour* for *seventeen scccessive hours* ; and we have heard authentically of a western physician, who emptied into the stomach of a patient laboring under billious remittent, *an ounce bottle* of s. quinine *in one night.* From thirty to fifty grains are now spoken of, as not unfamiliar doses, and even one hundred grains are occasionally given at once, and, we are assured, both with safety and striking success. It is in France, however, that the largest amounts of this salt have been employed. Guersent and Revillon speak of a Dr. Bazire, as having been remarkable for his enthusiastic and exclusive confidence in quinine as a remedy for the violent and pernicious intermittents of the department in which he practiced. Madame Bazire being seized with the prevailing malaria fever, took from him " in a very short space of time," 240 grains (16 grammes) of s. quinine. Soon after, the symptoms increasing, he gave her at one dose, 375 grains (25 grammes) more than three fourths of an ounce. At this juncture, fortunately for her, he fell sick, and the care of her devolved on other hands. He hastened to administer to himself, by the mouth and by the rectum, 900 grains of the sulphate of quinine, (60 grammes,) nearly two ounces, in a very short space of time ; and still further took during the space of eight or nine days, five ounces. He died a martyr to his reckless and implicit reliance on the drug ; and she recovered imperfectly, having been for a long time, both deaf and blind, the senses both of sight and hearing still remaining feeble."

The opinion of Dr. B. as to the dose in which the Quinine will be liable to produce fatal results, is contained in the following paragraph :—

" From all that I can gather, I am disposed to think from fifty to eighty grains of a pure article of quinine, given in solution at one dose, will produce death, nine times out of ten, in healthy .adults, and occasionally even smaller quantities. How far its operation may be modified by morbid action is a matter of consideration at the bedside.

We conclude our analysis with the following extract, which contains significant and important considerations:—

"I will not accuse any member of the profession of a want of common honesty in not reporting the fatal cases from the poisonous effects of quinine, which may have occured under his observation, but all the circumstances constrain me to believe that many results of this kind have occurred of which the profession have never been advised, and that it has often been the cause of injury and even death, when its agency has not been recognised, especially in the hands of those who were prepared only to witness its salutary operation. A fatal case is reported in a number of one our medical periodicals as descriptive of a form of congestive fever, which it is alleged the quinine *failed to cure*, but which, to my mind, *it most clearly produced.* The valuable paper by Professor S. H. Dickson, so often referred to here (*Southern Journal of Medicine and Pharmacy,*) contains some valuable hints touching this subject. In one paragraph he says' " look for its injurious operation upon the human system, as displayed in a set of phenomena closely resembling the series of symptoms which characterize the very class of fevers in which it is most clearly indicated," &c. And, when it is said of the unfortunate Dr. Bazire, that " il voyait avec effroi la maladie triomphante, et la puissance de son remide, qu'il croyat infaillible, trop souvent inefficace," who can doubt, after knowing the doses in which he employed it, that this " power of his remedy" *produced* the very state of things which he blindly persisted in believing it would relieve if only given in sufficient quantities, until, with this wild, though honest conviction, he doubled for himself the already fatal dose. Perhaps in other places, as well as in Martainville,* we would have fewer cases of "*malignant fevers*" if quinine was used with more moderation.

The injunctions of cautiousness in the use of quinine, which Dr. Baldwin's paper is calculated to enforce, are, in our opinion, timely and judicious. That this drug possesses remedial powers not as yet adequately estimated by a considerable portion of the profession, we firmly believe; but, we are not apologists for the large doses that some practitioners profess to employ. Several years siuce we had an opportunity of making some experimental observations on the effects of quinine in larger doses than were used at that time, or that we had known to have been administered. With not a little hesitation and anxiety we ventured from ten grains gradually to thirty, and, in one instance, to forty grains. The cases were of simple intermittents, affording a pretty good field to determine not only the anti-periodic virtues of the remedy, but its immediate, appreciable effects on the system. Our object was, in part, to ascertain the limit within which its remedial properties may be safely employed. The experiment of forty grains determined us never to repeat it, and we arrived in our own mind at the conclusion, that twenty-five or thirty grains, was the extent to which it might be properly administered in a single dose, or in divided doses with short intervals. We believe that under any circumstances all that the remedy is capable of effecting can be secured by doses within the limit just mentioned, and, ordinarily, by a quantity considerably less. On this subject our experience and reflection lead us to concur fully with the views of the author of the paper, an analysis of which has been given.

The remarks contained in the extracts last quoted might be extended in their application so as to embrace the use of other medicinal substances. The tendency to heroic practice, and the *nimia diligentia*, are to be deprecated, first and chiefly, on account of the direct evils arising from their pernicious consequences to life and health. But, aside from this, they prevent us from acquiring true knowledge of diseases, and a just appreciation of the recuperative forces. An authentic natural history of disease divested of all extrinsic influences, would be one of the most interesting and instructive of books; but such a book can hardly be written. Homœopathy might perhaps furnish it were its records to be relied upon as the records of disease honestly treated after the homœopathic system, and were Homœopathists both competent observers, and honest historians. As it is, it can only be expected to supply some fragments, which should not be overlooked by the medical philosopher. Let us not be understood by these remarks to disparage medicine. To admit that medical science is far from having attained perfection in the scope and accuracy of its principles, that the exercise of a sound and disciplined judgment is requisite to secure against serious practical errors, and that remedial agents are capable of being abused, is to concede nothing to the prejudices of the few who would depreciate its validity and usefulness. Medicine can afford to confess that it is a progressive science, constantly advancing as we believe and know, but, yet, far from that point at which its utmost capabilities will be realized. It is for the ephemeral systems which thrive for a brief period upon the credulity and ignorance of mankind, to assert claims of having attained all that is attainable. Such arrogant pretensions are soon discovered to be false, but this unfortunately does not prevent the rise of new delusions upon the ruins of those which have had their day and are forgotten. The *marvelousness* of a certain portion of mankind is never exhausted, but seems to increase by each successive disappointment, until novelty alone is a sufficient recommendation for the transient currency of any new absurdity or fraud.

But to return from this digression, it often happens that the tendency of the mind to oscillate between extremes, results in the final neglect or abandonment of valuable remedies. Instances in illustration will occur to the minds of every practitioner. Believing, as we do, that the Quinine is possessed of very great and extensive powers in controlling certain morbid actions, we hope that its use will be such as more and more to define the nature and extent of these powers, and not to develop evils, which, by favoring the prejudices of the public, and creating in the minds of physicians an undue timidity in prescribing it, will lead to the curtailment of its safe and useful availability.

